# Aids to Nursing

**Published:** 1888-04-07

**Authors:** M. M. Basil

**Affiliations:** Author of "The Commoner Accidents to Life and Limb.


### Aids to Nursing.

By M. M. Basil, M.A., M.B., C.M., Author of "The Commoner Accidents to Life and Limb."

*(Continued from p. 442, vol. iii.)*

### Food and Feeding.

In disease it is advisable to watch and find out what a patient's fancies are with regard to food, as sometimes this knowledge may be of help in determining his diet. In convalescence, on the other hand, his appetite is no standard to the quality or quantity of food required. Never satisfy the cravings of a convalescent patient, especially after fevers. The clearly expressed and persistently repeated wishes of the patient himself on the subject of the diet suitable for him cannot always be ignored; you must, therefore, report the expression of such longings to the doctor. But when once you have specific directions as to dieting, you should not in the least yield or be carried away by the popular notion of consulting the longings or whimsical cravings of the patient.

"A sick man's appetite who desires most that
Which would increase his evil—"

was pointed out long ago as a dangerous guide to be relied upon. Unless you observe this rule unflinchingly you will be guilty of some of the murders or very questionable forms of homicide which are committed repeatedly with chops, steaks, pickles, cheese, oranges, greens, etc. "There is death in the pot" for many patients suffering from typhoid fever, dysentery, peritonitis, and chronic diarrhœa. The dieting must be unflinchingly strict, particularly in chronic dysentery. It is well to remember that many of these last cases are prolonged by the use of whole-meal bread, in which the irritating and tough outer coverings of the grain are found in abundance.

In all these cases the self-control of the patient must not be tested in any way, as it is certain to fail. His statements as to the kind of food taken in secrecy must also be received with suspicion. The assurances of the dysenteric patient regarding this point can no more be trusted than the solemn pledges of the chronic tippler.

You will find that many of the best-intentioned friends of the patient will elude your vigilance and do him some murderous kindness. Keep a sharp look-out on the patient and his friends, and save him from such dreadful enemies. In cases where nourishing food will be necessary for days together, it is best not to accustom the patient to delicacies such as iced drinks, lemonade, etc. The patient, in these circumstances, soon learns to refuse milk, and thus adds to the difficulties of the treatment.

Many patients, especially in acute diseases, suffer much from thirst. In all countries the sick among the poor are subjected to fearful cruelty owing to the popular dread of cold water in very feverish conditions. You should have no scruples in allowing your patient to drink cold water with moderate freedom, unless ordered to the contrary. Perhaps, in the severer forms of inflammations of the stomach and bowels, it would be better to withhold cold water until especial directions are got on the subject; but in all other cases use it freely in small quantities. Remember to offer only so much water to the patient as you wish him to drink. It is needless cruelty to give a whole tumblerful of water to a feverish child, and then attempt to wrench it off its hands after the first mouthful.

When no water can be given by the mouth, owing to severe vomiting or serious stomach disease, the distressing thirst so often present can be quenched by gently injecting large quantities, *e.g.*, a pint, of warm water into the bowel. The water, under these conditions, is rapidly absorbed wholly or in part. As we shall see later on, this is an exceptionally large quantity of liquid for an enema, which is specially meant to be retained by the bowel.

Before concluding this subject I must ask you to lose no opportunity to learn good cooking. The chief chemical principles of the subject, as well as the various little artifices of an accomplished artist should be familiar to every good nurse. Cooking, it would appear from history, was at one time a necessary portion of women's education; but under the influence of civilisation and high-pressure mental culture it is disappearing from societies farthest removed from the primitive long-tailed condition. Travellers, however, often make remarks on the subject almost subversive of one's complete faith in some of the principles which guide this enlightened century. Thus, for instance, speaking of English cooking versus Indian cooking, Dr. Norman Chevers says: "The philosopher who defined man as 'a cooking animal' certainly had not in view our otherwise excellent sisters the women of England." Such a statement could be made only by one enjoying the privileges usually allowed to a traveller.

"Not on Morality, but on Cookery let us build our Stronghold," said Carlyle, in one of his dyspeptic and biting moods. Perhaps the Hebrew historian who described Isaac as incapable of blessing his first-born with fervour enough until after a good feed of savoury dishes, knew more on the subject of the civilizing influence of the kitchen on the human soul, not excepting the intellectual powers and moral faculties. Certainly the sum-total of annoyances caused by alcoholic intemperance, dyspepsia, irritable temper, the heart-ache and the thousand natural shocks that flesh, such as poor Mrs. Caudle's, is heir to, can be materially diminished in modern society by greater attention to the preparation of food. The kitchen hath verily charms to tame the savage breast. And when at your wits' end to find the best place to apply soothing remedies to the irritable heart, you may remember with profit that, anatomically, the nearest way to the heart is through the stomach.

The duties of the nurse in the sick room may be roughly divided into two classes: first, her work as an assistant in enabling the medical man to ascertain the nature of the disease and record its progress; and secondly, her work as an agent in ministering to the wants of the patient and aiding the process of recovery by various remedial measures. In the order of time the first set of duties must naturally precede the second, but for purposes of convenience we shall reverse this arrangement, and take up the consideration of the second group before that of the first.

### Baths.

The different forms of baths rank among our aids to recovery from disease. They are also important agents for warding off many of the conditions which make us liable to attacks of disease. Their consideration comes naturally before that of the various hot and cold applications and dressings. A warm bath may be looked upon as a poultice applied to the body generally. With less truth in the analogy, a cold bath may be viewed as a similar application to the entire surface of the body. The baths which are most commonly used in disease are the tepid and the warm bath. These are used principally for purposes of cleanliness; they also produce a soothing effect, and aid the action of the skin very materially. In all well-regulated establishments a warm bath is given to each patient soon after admission and at regulated times subsequently, according to the nature of the case. Some people look upon all sickness as a Providential arrangement designed to bring men's minds back to God. The view certainly has its comforts and uses, and he would be a bold man who could say that in no case is it true. You should, at any rate, give the patient the benefit of all the advantages which sickness brings with it, and make it a time of purification of the outer temple. The mind will then be all the more disposed to avail itself of such hidden blessings as may lie behind disease. You will meet some patients who have decided objections to parting with their "artificial surfaces;" indeed, a few would almost prefer having their grinders pulled out of their head to getting the thirty miles of carefully-constructed tubing which Nature has laid on in their skin put into a sanitary condition.

A considerable amount of discretion must be exercised in the selection of cases fitted to bear even a tepid bath. In all cases of doubt wait for medical sanction. Some of the conditions in which you should be particularly careful are—dropsical cases, all patients who appear to have cardiac or respiratory disease, and all weak patients, especially if very young or old. It is not expected of you to make out all these conditions exactly, but with care and observation you will learn to suspect the existence of danger, and your patient should always have the benefit, not only of the doubt, but even of mere suspicion. No nurse would, of course, think of placing a case of severe injury into a bath except when especially instructed to do so. Cases of paralysis, even when old standing, require extreme care in the regulation of the temperature of the bath. Paralysed limbs are very sensitive to slight ranges of temperature, and there is always risk of having a repetition of the attack if there is any undue excitement of the general condition of the patient.

If a patient is exhausted, paralysed, or subject to any form of fits, you must never leave him unattended even for a fraction of a second. Should he faint or get into a fit, complete immersion may take place with consequent death. Even a shallow bath may prove dangerous: the patient may turn round in a fit, and be suffocated.

I shall quote a passage from one of the most charming of recent contributions to medical literature to impress on you further that in the different baths we have very ticklish two-edged weapons, so that good or harm may be done according to the amount of attention paid to the subject: "A man, whom I could not detect to have any heart disease, once died in my hands, as it were, when I was sitting beside him, after being less than an hour in water at 103° F. . . . I know of two other cases where syncope and death resulted in the same way. . . . I now prefer 99° F. as the proper temperature."

Before commencing to get a bath ready, you should attend to the general temperature of the room. Draughts in it must, above all, be prevented. You must have everything in readiness, so that after the bath you can get the patient placed quickly in bed or before a good fire. With ordinary care all colds in this condition can be prevented. The exaggerated popular dread on the subject must be classed with the ghost stories of the nursery tale series. Always time yourself before putting a patient into the bath.

*(To be continued.)*

